# Effect of Tongue Thrust Swallowing on Position of Anterior Teeth

**DOI:** 10.5681/joddd.2009.019

**Published:** 2009-09-16

**Authors:** Tahereh Jalaly, Farzaneh Ahrari, Foroozandeh Amini

**Affiliations:** ^1^Associate Professor, Department of Orthodontics, Faculty of Dentistry and Dental Research Center, Mashhad University of Medical Sciences, Mashhad, Iran; ^2^Assistant Professor, Department of Orthodontics, Faculty of Dentistry and Dental Research Center, Mashhad University of Medical Sciences, Mashhad, Iran; ^3^Dentist, Private Practice, Mashhad, Iran

**Keywords:** tongue thrust, incisor teeth, overjet, overbite, cephalometry

## Abstract

**Background and aims:**

There is no consensus about the effect of tongue thrusting on incisor position. The purpose of this study was to evaluate the position of anterior teeth in growing children with tongue thrust swallowing.

**Materials and methods:**

In the present study 193 subjects with an age range of 9 to 13 years participated. All the patients were examined by a trained investigator and those having tongue thrust swallowing were selected and the position of their anterior teeth was compared with a control group consisting of 36 subjects with normal occlusion. Data was analyzed by independent sample t-test.

**Results:**

Among the 193 students who were examined in this study, 10 cases (5%) were diagnosed to be tongue thrusters. Overjet was significantly increased in tongue thrust individuals (P < 0.05), while the other variables were not statistically different from the controls (P > 0.05).

**Conclusion:**

The results indicated that tongue thrust may have an environmental effect on dentofacial structures. Considering the high incidence of tongue thrust in orthodontic patients, it is suggested that dental practitioners observe patients of all ages and those in all stages of orthodontic treatment for evidence of tongue thrust swallowing.

## Introduction

The relationship between form and function of the stomatognathic system has been evaluated by many investigators. It has been suggested that the size,^1 - 5^ function ^6 - 8^ and posture ^9 - 11^ of the tongue might have some effects on the surrounding oral environment. However, it has long been debated whether tongue function would lead to malocclusion or it merely adapts to local changes of occlusion.^2 , 12 - 14^, Although some investigators consider the size and dysfunction of the tongue as essential etiological factors in the development of malocclusion,^11 , 15 - 17^ others believe that tongue thrust swallowing should be considered a result rather than the cause of malocclusion.^9 , 18 , 19^ Their rationale is that in the presence of overjet or open bite, it is difficult to seal off the front of the mouth during swallowing. According to Proffit^10^ the tongue thrusts forward to gain anterior valve function in order to prevent the escape of food or liquids. However, the reverse is not always true. A tongue thrust swallowing is often present in children with good anterior occlusion.

A wide range of tongue thrust incidence has been reported in the literature. Tulley^20^ reported an incidence of 2.7%, while Bell and Hale ^21^found 74% of children in grades 1 through 3 to be tongue thrusters. It has been shown that the incidence of tongue thrusting is higher than normal in subjects with open bite or overjet malocclusions.^22 , 23^Tongue thrust swallowing has been assumed to be a contributing factor in the relapse of treatment results.^22 , 24 , 25^ Many research studies have pointed out that a significant percentage of relapse after orthodontic treatment might be related to orofacial muscle imbalance and deviated swallowing. Fotis et al ^26^ observed that a dental relapse as a result of skeletal relapse is seen only in cases in which normal perioral function, including normal lip closure and absence of tongue thrust swallowing has not been established after orthodontic treatment. Ozbek et al ^27^ reported that in patients with excellent retention of maxillary expansion, the tongue may spontaneously position itself closer to the palatal roof, thus counteracting buccal pressure.

The effect of tongue thrust on dental and skeletal morphology has been evaluated in several studies. It has been demonstrated that protrusive tongue activity (tongue thrust) during swallowing might result in labial inclination of incisors, open bite and spacing problems in some cases.^6 , 28^Overstake^28^ concluded that there is a functional relationship between deviated swallowing and open bite as well as overjet.However, some authors believe that the total duration of swallowing in a normal subject is too short to produce morphological changes.^10^ There is no consensus about the effect of tongue thrusting on incisor position and the influence is not quite clear in growing children. The purpose of this study was to evaluate the position of anterior teeth in growing children with tongue thrust swallowing.

## Materials and Methods

Of 526 subjects who were referred to the Department of Orthodontics at Mashad Faculty of Dentistry, 193 children (99 girls, 94 boys) were selected and evaluated for evidence of tongue thrust swallowing. Inclusion criteria consisted of patients with an age range of 9 to 13 years, Angle class I occlusion, and complete eruption of upper and lower incisors. Individuals with craniofacial deformities as well as subjects who had undergone orthodontic treatment and those having oral habits were excluded from the study. Informed consent was obtained from the parents of the subjects after a brief explanation of the study.

All the patients were examined by a trained investigator and those having tongue thrust swallowing were selected and anterior teeth positions were compared with a control group consisting of 36 subjects (18 girls, 18 boys, average age 11.2 2.1 years) with Angle class I occlusion, normal overjet and overbite, and normal sagittal and vertical skeletal relationships. None of the controls had any soft tissue abnormality or respiratory problems.

Data on tongue thrust swallowing was obtained at the time of clinical examination. In order to examine the presence of tongue thrusting, the patients were asked to swallow their saliva three times during the same visit. When in doubt, another swallow was requested until the dentist was satisfied with his judgment. Tongue thrust was defined as protrusion of the tongue between upper and lower incisors or cuspids during swallowing.

###  Measurements

The usual orthodontic documentation, including dental casts and cephalometric radiographs, were obtained for the subjects. The records of control subjects were available in the archives of the Orthodontics Department. Overjet and overbite were measured to the nearest 0.1 mm directly from the dental casts using calipers. The casts were trimmed using a wax recording of subject’s bite at the time of examination. Overjet was measured in millimeters as the difference between the incisal edge of the most proclined upper anterior tooth and the corresponding point on the labial surface of the mandibular incisor. To measure overbite, the incisal edges of upper anterior teeth were marked on the labial surface of lower anterior teeth and the distance between the incisal edges of lower incisors to the mark gave overbite in mm.

**Figure 1:**
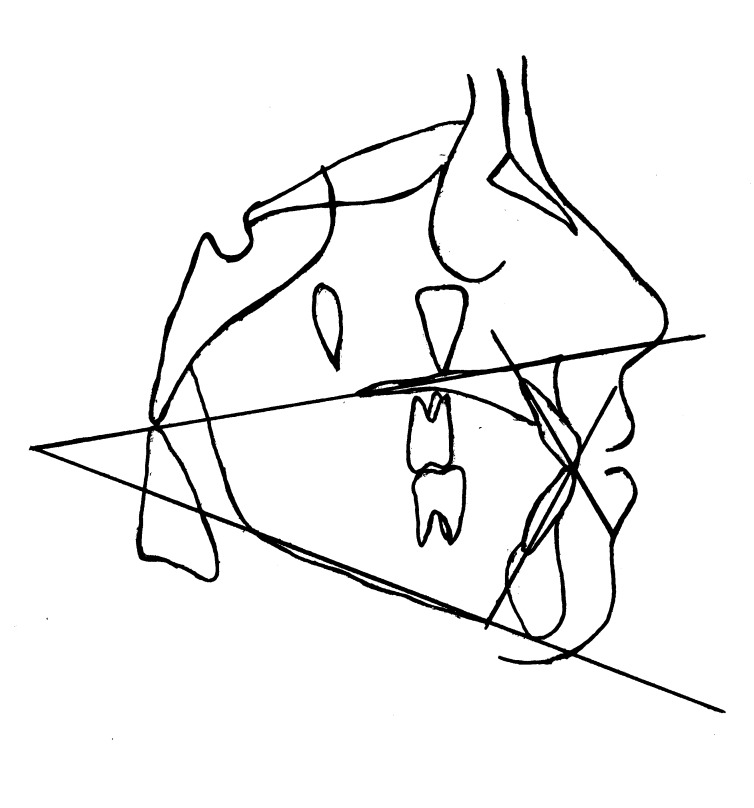
Cephalometric reference points and planes used in the present study.

Radiographs were obtained in NHP at the Radiology Center of Mashhad Faculty of Dentistry. The subjects were asked to keep their teeth in centric occlusion with the lips relaxed. Patients were instructed to hold their breath and not to swallow while the radiographs were taken. The cephalograms were traced by one investigator and the accuracy of landmark identification was confirmed by another investigator. To minimize the error caused by head positioning, the midline of double contour bilateral structures was drawn. Three angular variables were measured to the nearest 0.5° on these tracings. The reference points and lines used in the cephalometric analysis are shown in Figure 1.

All the measurements were carried out by the same investigator to eliminate interexaminer variability. The reliability of the measurements (intraexaminer errors) was examined by re-measuring dental casts and cephalograms of 10 randomly selected subjects by the same examiner with a 1-week interval. The error of measurements was calculated as 0.2 mm and 0.5 degrees for linear and angular measurements, respectively, using Dahlberg formula.

### Statistical analysis

The overjet, overbite and cephalometric variables of tongue thrusting individuals were compared with the corresponding values of control subjects, using independent samples t-test. Significance level was set at α < 0.05.

## Results

Among the 193 students who were examined in this study, 10 cases (5%) were diagnosed to be tongue thrusters. Descriptive statistics of the variables analyzed in the test and control groups and a statistical evaluation of intergroup differences are given in Table 1. Comparison of dental features between tongue thrusting and control subjects showed that overjet was significantly greater in tongue thrusting individuals (P < 0.05), while the mean overbite, upper incisor inclination, lower incisor inclination and interincisal angle were not statistically different between the two groups (P > 0.05).

**Table 1:** Position of anterior teeth in tongue thrust subjects

	Tongue thrust group (n=10)		Control group		
Measure	mean	SE	mean	SD	P value
Overjet	4.3	0.373	1.66	0.85	0.005
Overbite	0.8	0.787	1.55	1.02	0.12
U1 to Pal-P	111.5	2.352	109	4.98	0.19
L1 to Man-P	94.5	2.552	91.5	4.54	0.32
U1 to L1	126	2.938	131	5.14	0.43

S denotes significant. NS denotes not significant.

## Discussion

The results of this study showed that overjet is the only variable which significantly increases in tongue thrust individuals compared to control subjects. Hanson et al ^29^ reported that the deleterious forces of the tongue result in excessive eruption of posterior teeth, open bite or overjet. There were no significant differences in overbite, upper incisor inclination, lower incisor inclination, and interincisal angle between the groups of this study. This finding is contradictory to the results of a study carried out by Alexander and Sudha^6^ who reported a significant increase in proclination of upper anterior teeth in tongue thrust individuals. It should be noted that the mean amount of overbite was smaller in the test group compared to control subjects and although the difference was not statistically significant, there were 3 cases of anterior open bite in the test group.

In the present study, subjects with at least 9 years of age were selected because at this age the anterior teeth of most children have erupted. Habitual swallowing of saliva was chosen rather than water swallowing, since a normal adult repeats this normal swallowing pattern between 1200 and 3000 times every day.^30^Therefore, swallowing of saliva might have a stronger effect on dentofacial morphology compared to water swallow-ing.

Increased overjet in the tongue thrust group implies that there may be a relationship between tongue function and dental morphology. Teeth are under a variety of forces including chewing, and the forces of the lips, cheeks and tongue. These forces, whether intermittent or continuous, are large enough to cause tooth movement.^10 , 11^ Some studies have shown that dental changes in tongue thrusters result from the increased electrical activity of the genioglossus muscle and the prolonged duration of swallowing in these subjects.^6^

The effect of tongue thrust on dentofacial development depends on several factors: the frequency of swallowing or how often the tongue exerts force on the teeth, the severity of the force exerted on the teeth, the counteraction of these factors by other muscular structures such as the lips, the resistance of dentoalveolar structures to displacement, and finally the resting posture of the tongue when no swallowing is occurring.^31^ The duration of swallowing has been reported in many previous studies. In an electromyographic investigation by Findlay and Kilpatrick ^32^ the average swallowing time was found to be about 2 seconds. Sonies et al ^33^ reported that the duration of swallowing was between 1.7 and 3.4 seconds for swallowing saliva. In a study carried out by Ichida et al^34^ the duration of lingual-palatal contact during saliva swallowing ranged from 1.1 to 2.9 seconds. It should be noted that tongue tip protrusion is sometimes associated with a low forward posture of the tongue. Even if the amount of force is very low, this can influence tooth position horizontally or vertically since the duration of force is long. It has been demonstrated that prolonged low tongue position during the growth period in children may result in excessive molar eruption causing a clockwise rotation of the mandible, a disproportionate increase in lower anterior face height, retrognathia and open bite. A low tongue position may also prevent lateral expansion and anterior development of maxilla.^2 , 35^

Although most of the variables evaluated in this study were not significantly different between normal subjects and tongue thrust individuals, the limitations of the study should be considered. The sample size in the tongue thrust group was relatively small. In addition, the large standard deviation of variables must be considered. This large variation in the value of each variable may be due to differences in duration, intensity or frequency of tongue thrust swallowing in different subjects.

It is important for orthodontists to understand the effect of tongue function in the correction of malocclusion and stability after treatment. It has been reported that tongue thrust may be initiated during orthodontic treatment, especially when treatment creates temporary open spaces or interferences with intercuspation or reduces tongue space.^36^ Cheng et al ^31^ proposed that all tongue dysfunctions should be corrected if long-term stability of treatment results is desirable. Myofunctional therapy is often indicated for correction of tongue thrust swallowing. It has been demonstrated that both myofunctional therapy and crib therapy are successful in correction of tongue thrust swallowing.^6 , 37 , 38^ However, Subtelny^19^ did not find any benefit for tongue thrust treatment.

Using a standard procedure, tongue thrust assessment can be achieved simply by practitioners, parents or teachers. With respect to the high incidence of tongue thrust in orthodontic patients, and considering the possible relationship between deviated swallowing and dentofacial morphology, it is suggested that dentists observe patients of all ages for evidence of tongue thrust swallowing.

## Conclusion

The results of the present study showed that tongue thrust may have an environmental effect on dentofacial structures. Considering the high incidence of tongue thrust in orthodontic patients, it is suggested that dental practitioners observe patients of all ages and those in all stages of orthodontic treatment for evidence of tongue thrust swallowing.
